# PlaneNet: an efficient local feature extraction network

**DOI:** 10.7717/peerj-cs.783

**Published:** 2021-12-07

**Authors:** Bin Lin, Houcheng Su, Danyang Li, Ao Feng, Hongxiang Li, Jiao Li, Kailin Jiang, Hongbo Jiang, Xinyao Gong, Tao Liu

**Affiliations:** 1 Sichuan Agricultural University, College of Information Engineering, Yaan, Sichuan, China; 2 Sichuan Agricultural University, College of Science, Yaan, Sichuan, China

**Keywords:** Feature extraction, Local feature fusion, efficiency, Strong operability, Reduce redundant

## Abstract

Due to memory and computing resources limitations, deploying convolutional neural networks on embedded and mobile devices is challenging. However, the redundant use of the 1 × 1 convolution in traditional light-weight networks, such as MobileNetV1, has increased the computing time. By utilizing the 1 × 1 convolution that plays a vital role in extracting local features more effectively, a new lightweight network, named PlaneNet, is introduced. PlaneNet can improve the accuracy and reduce the numbers of parameters and multiply-accumulate operations (Madds). Our model is evaluated on classification and semantic segmentation tasks. In the classification tasks, the CIFAR-10, Caltech-101, and ImageNet2012 datasets are used. In the semantic segmentation task, PlaneNet is tested on the VOC2012 datasets. The experimental results demonstrate that PlaneNet (74.48%) can obtain higher accuracy than MobileNetV3-Large (73.99%) and GhostNet (72.87%) and achieves state-of-the-art performance with fewer network parameters in both tasks. In addition, compared with the existing models, it has reached the practical application level on mobile devices. The code of PlaneNet on GitHub: https://github.com/LinB203/planenet.

## Introduction

Convolution neural networks (CNNs) have achieved excellent performance in image classification, semantic segmentation, and object detection tasks. Due to the limitations of computer resources, the scale of CNNs is restricted. With improved computer performance, deeper CNNs, where the CNN structure becomes more complex with many parameters and floating-point arithmetic operations, are needed to improve the precision of computer vision tasks. For example, ResNet50 has approximately 25.6M parameters and requires 4.1B Madds to process an image of shape 224 × 224 (Han et al., 2020).

A deep convolutional neural network is composed of many convolutions, and its scale is often represented by two factors: the number of channels and the number of layers. By deepening CNNs, smaller convolutional kernels are generally used, and the calculation can be further reduced by pooling, such as with VGG networks (Simonyan & Zisserman, 2015). As a network deepens, the extracted final features become more abstract. Therefore, the features learned from the previous layer are combined by increasing the number of convolution kernels and using skip connections to fuse the various layers, such as with ResNet networks (He et al., 2015). The deepening of the network improves the accuracy to some extent, but it does not necessarily lead to a good trade-off between the speed and the scale of the network. Moreover, complex networks will experience a significant increase in the calculation and floating-point operations of the model, which is not conducive to the application of models on mobile terminal devices and embedded devices.

In recent years, with the rapid development of the Internet of things, the trend of deep neural networks is designing a lightweight network that simplifies the model while achieving comparable accuracy, which can reduce the demand for computer hardware resources. There are many efficient deep neural networks with fewer parameters and computations. For example, SqueezeNet (Iandola et al., 2016) decreased the number of channels to reduce the size of the model. The depthwise convolution introduced by the MobileNet series helps the model make a good trade-off between training resources and accuracy (Howard et al., 2017; Sandler et al., 2019; Howard et al., 2019). In particular, MobileNetV2 is very representative. The dimensions of the input feature are increased by using the 1  × 1 standard convolution followed by the 3  × 3 depthwise convolution. Finally, the dimensions of the features are further reduced with the 1  × 1 standard convolution operation. The advantage of this setting is that the number of channels can be increased, and more features can be obtained. However, excessive standard convolutions make the information of a single pixel in the channel of the network constantly fused and even redundant and reduce the information interaction in the plane region considering the image. However, due to the expensive dense 1  × 1 convolution, state-of-the-art basic architectures such as Xception (François, 2017) and ResNet become less efficient in tiny networks. This results in significant increases in the number of parameters and Madds. ShuffleNet completely correlates input and output channels by sparsely connecting them in group convolution and uses residuals and pointwise to replace the 1  × 1 convolution (Zhang et al., 2017; Ma et al., 2018). EspNetV2 (Mehta et al., 2019) uses the deep dilated separable convolution and replaces the additive feature fusion method with concatenation to increase the dimension of features. By fusing the down-sampling information of the original input image, the feature information becomes richer. GhostNet introduced a ghost module for feature graphs in a more cost-effective way while retaining redundant feature graphs. Besides, pruning, network architecture search (Tan et al., 2018), knowledge distillation, and other methods also play essential roles in lightweight model design.

Based on these observations, we believe that the 1 × 1 convolution is crucial in a lightweight network. Using the 1 × 1 convolution more reasonably can effectively improve the accuracy of a model and reduce the negative effects of the numbers of parameters and floating-point operations caused by redundancy. Based on this principle, we make more effective use of the 1 × 1 convolution by adjusting the position of the 1 × 1 convolution and setting the stride in the deep separable convolution and propose our network, named PlaneNet. In summary, our contributions are as follows:

1. We make more effective use of the 1 × 1 convolution, which improves the accuracy while reducing the unnecessary redundancy; and provide a practical reference for subsequent efficient network design.

2. Plane local features are the key elements of image classification. Increasing the information interaction of plane regions and effectively extracting the local features of images can help models understand the heart of models more effectively. We proposed a novel lightweight network PlaneNet, whose dimension is increased by the separable convolution, and then the local features are extracted more effectively by the 1 × 1 convolution. Finally, the plane local features are extracted from the data with spatial dimension features by using the separable convolution.

3. In our experiments, our model can achieve state-of-the-art performance in classification and semantic segmentation tasks.

The organizational structure of the remainder of this article is as follows. We begin with ‘Materials & Methods’ that describes the principles and methods used by PlaneNet. In ‘Results’, we compare our model with other mainstream lightweight networks. In Section 4, we discuss the experimental results of our model and consider the advantages and disadvantages of the improved strategy of our model. ‘Conclusion’ contains the conclusions and future work.

## Materials & Methods

This section introduces the plane module taking the MobileNetV2 module as a representative for a detailed comparison. Then, the analysis of two critical factors and the complexities of the plane module is demonstrated. Finally, PlaneNet is developed.

### The importance analysis of 1 × 1 convolution

To prove the importance of the 1 × 1 convolution in the model, experiments are conducted in ‘Hyperparameter Strategy on CIFAR-10’. For example, the CIFAR-10 experiment, an extreme example, assumes that local plane characteristics are not considered and fully uses the 1 × 1 convolution kernels for the standard convolution operation. The result is calculated using 22.29% of the time. The remaining 1 × 1 convolution kernels form a set of complete traditional convolution networks with 90% accuracy; therefore, extracting plane local feature from the network has a crucial role.

The essential functions of the 1 × 1 standard convolution are to extract the depth pixel features and change the number of channels, but the disadvantages are obvious. The number of parameters is still large, and the receptive field is tiny. The 1 × 1 standard convolution in MobileNetV1 accounts for 95% of the computation time and contains 75% of the parameters. Therefore, we need to seek a strategy that can consider both. Thus, the overall strategy guiding our designed module has two points:

**Strategy 1**: Within a specific budget of computing resources, the network should extract as wide a range of regional features as possible to richer network features.

**Strategy 2**: The network should reduce the redundant 1 × 1 standard convolutions so that the network, based on the effective use of depth extraction features, can also reduce unnecessary calculations to a certain extent.

### Plane bottleneck

**Figure 1:**
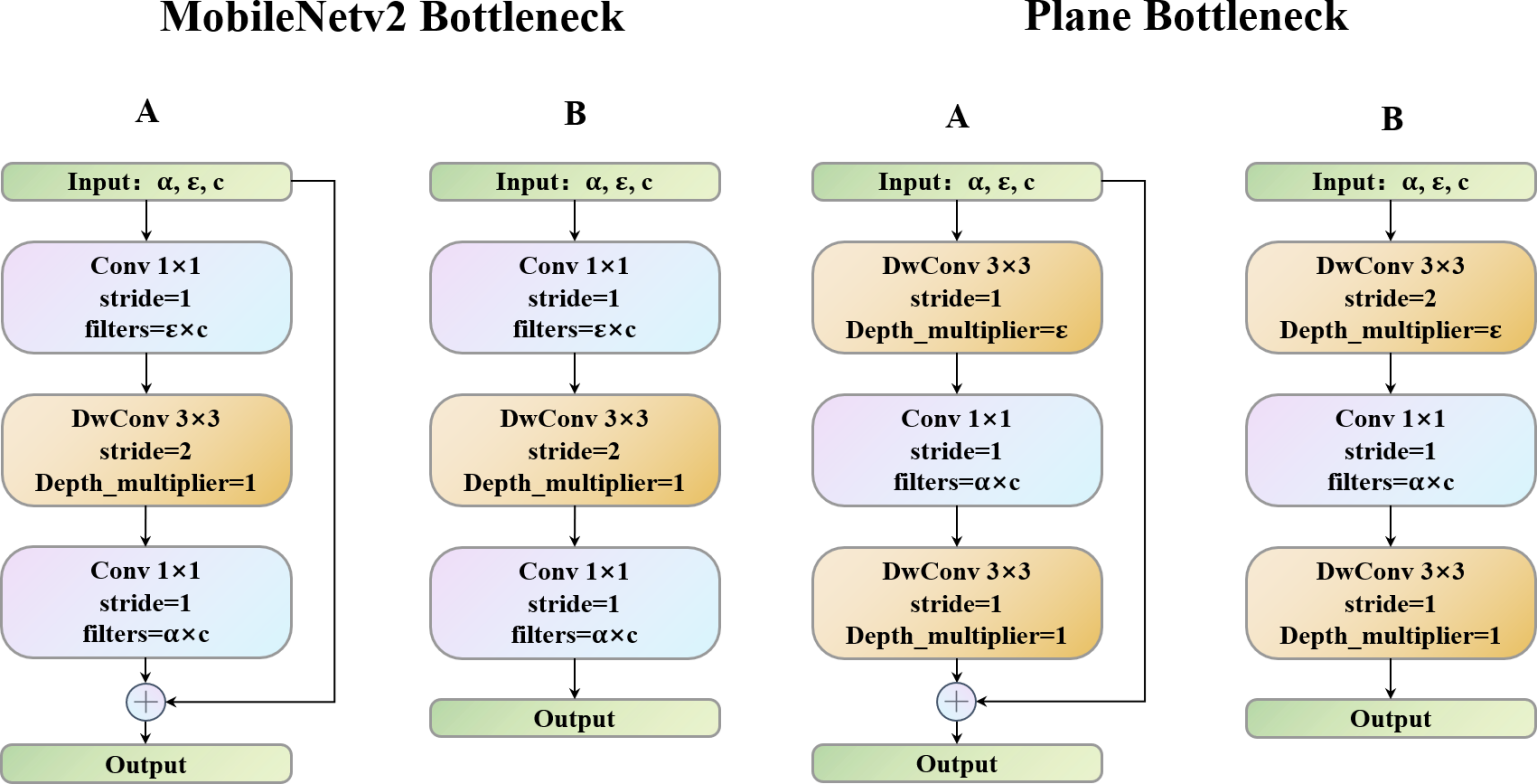
MobileNetV2 bottleneck *vs.* Plane bottleneck.

Deep convolutional neural networks usually consist of many different-sized convolutions, resulting in high computational costs. Since the SqueezeNet after the 3 ×3 convolution is replaced by the 1 × 1 convolution, the efficient network model design adopts the 1 × 1 convolution to extract local features. Then, different methods, such as the ShuffleNet group convolution and EspNetV2 that replace the point-by-point convolution with the grouping point convolution, are used to reduce the redundancy. All methods eased the partial feature extraction due to redundancy of the 1 × 1 convolution to a certain extent. Therefore, to more effectively use the 1 ×1 convolution, we propose the plane bottlenecks and use them to extract the features. As shown in Figs. 1A and 1B, in the right two modules, unlike MobileNetV2, through the depth of the dimension of the separable convolution layers, the depth of the 1 × 1 convolution operation will not only largely consider the image plane area’s information interaction but also use the cheap linear operation to enhance features, therefore meeting the requirements of strategies 1 and 2. In addition, in bottlenecks with a stride is 2, we use the residual block (Hariharan et al., 2011).

The point-by-point convolution follows the separable convolution, that is, the simple 1 × 1 standard convolution (Qin et al., 2018). The function of this layer is to conduct information interaction on the channel dimension of the data and make it fuse information on a single-pixel point. However, it is worth noting that, unlike MobileNetV2, the number of parameters in our 1 × 1 convolution is dramatically reduced. This is because the stride in the separable convolution is set to s, the image size is reduced, and the computational effort is reduced accordingly. The Madds of the point-by-point convolution after separable convolution is calculated as follows (the introduction of the parameters is given in ‘Materials and Methods’). D_k_ is the image size, and D_k_ is the size of the feature map. M is the number of input channels. N corresponds to the primary function used to change the number of channels in the output.

(1) \begin{eqnarray*}{D}_{f}\cdot {D}_{f}\cdot N\cdot M\cdot N\cdot M\end{eqnarray*}

The Madds of the ascending dimensional point-by-point convolution (both the input dimension and the output dimension) is calculated as follows:

(2) \begin{eqnarray*}{D}_{k}\cdot {D}_{k}\cdot N\cdot M\cdot N\cdot M\end{eqnarray*}

We can reduce the proportion of the calculation formula as

(3) \begin{eqnarray*} \frac{{D}_{f}\cdot {D}_{f}\cdot N\cdot M\cdot N\cdot M}{{D}_{k}\cdot {D}_{k}\cdot N\cdot M\cdot N\cdot M} = \frac{1}{{s}^{2}} \end{eqnarray*}

This result is further verified in ‘Compared with MobileNetV2 on Caltech-101’. Therefore, this design almost meets the performance requirements of the MobileNetV2 module design, and the required computing resources are significantly reduced. It can handle cross-channel features without the lack of interaction of plane local features.

**Figure 2:**
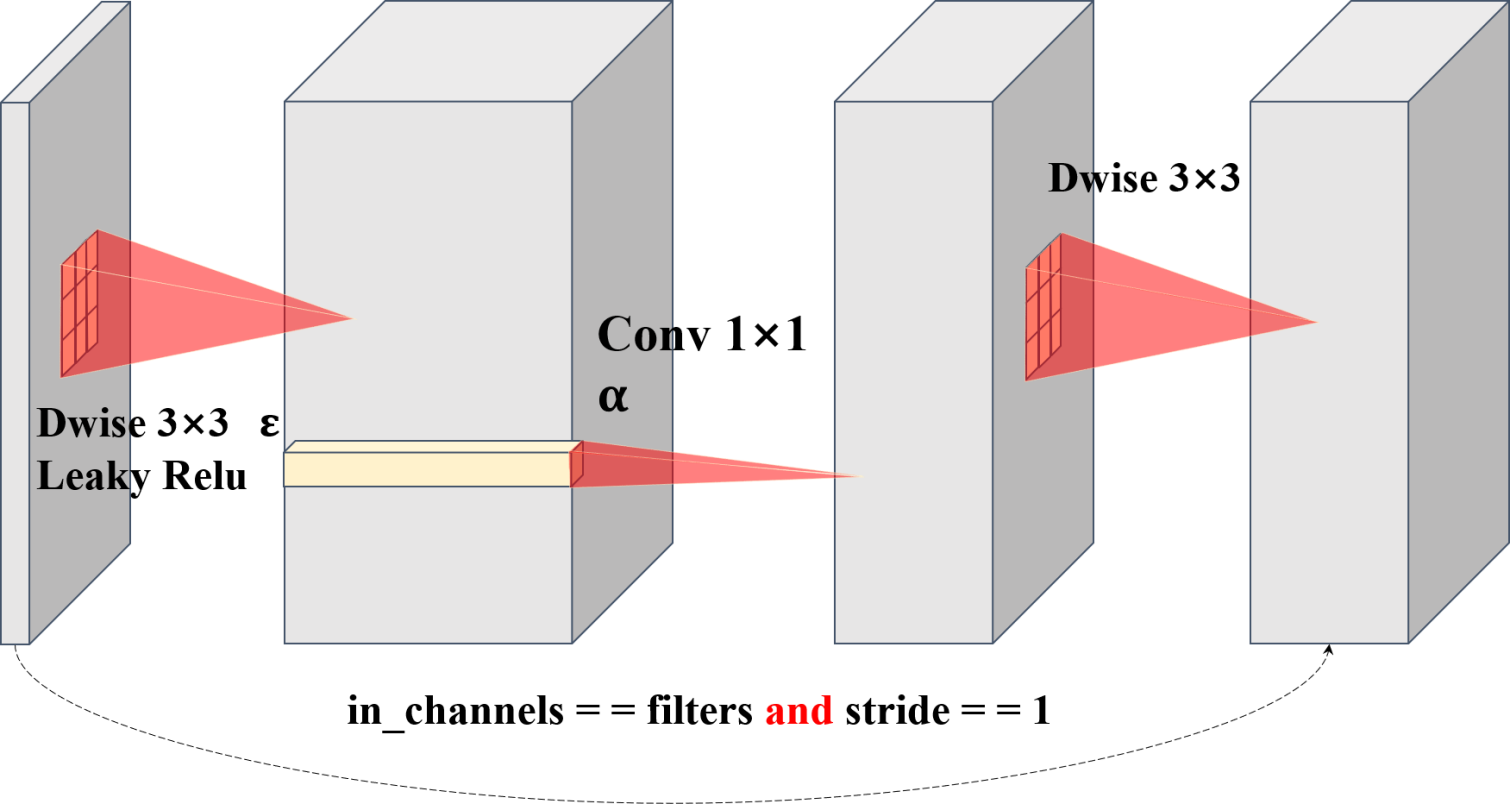
Structure diagram of plane bottlenecks. The dotted line represents a shortcut path. The shortcut path is adopted for stride = 1. For stride = 2, the shortcut path is removed.

Through our separable convolution and point-by-point convolution, we achieve the ascending dimension function of direct point-by-point convolution and introduce the information interaction between the local regions of the plane to increase its receptive field, which achieved by direct point-by-point convolution (Chen & Su, 2019; Sinha & El-Sharkawy, 2020; Sinha & El-Sharkawy, 2019). Plane bottlenecks are often used in similar bottleneck layer structures, so separable convolution is reintroduced to extract the local plane features of data obtained from spatial dimension features. The different convolution kernels of two separable convolutions perfectly address strategy 1 and strategy 2. For large-size images, two depthwise convolution kernels can coordinate with each other, setting different sized convolution kernels to increase the receptive field, and using different sized convolution kernels to obtain different receptive fields makes plane bottlenecks more operable and diverse. It is noteworthy that although the size of the convolution kernel can be artificially regulated, regulating the plane module seldom has many effects on the number of parameters in the network, as well as on the Madds. This means that our plane bottlenecks are much more flexible, decreasing the convolution kernel size by two when handling a small image and increasing the size of a large image. However, with the MnasNet, MobileNet, GhostNet, and ShuffleNet series models, we cannot find bottlenecks in which two separable convolutions are used to extract local features. Plane bottlenecks are a famous practice for retaining unique feature mappings and adding new fusion features (Sinha & El-Sharkawy, 2019). Plane bottlenecks take excellent account of in-plane feature fusion, which is why we call them plane bottlenecks. In ‘Hyperparameter Strategy on CIFAR-10’, we will continue to explore the effects of the size of different convolution kernels on plane bottlenecks. A complete schematic of plane bottleneck modules is shown in Fig. 2.

**Figure 3:**
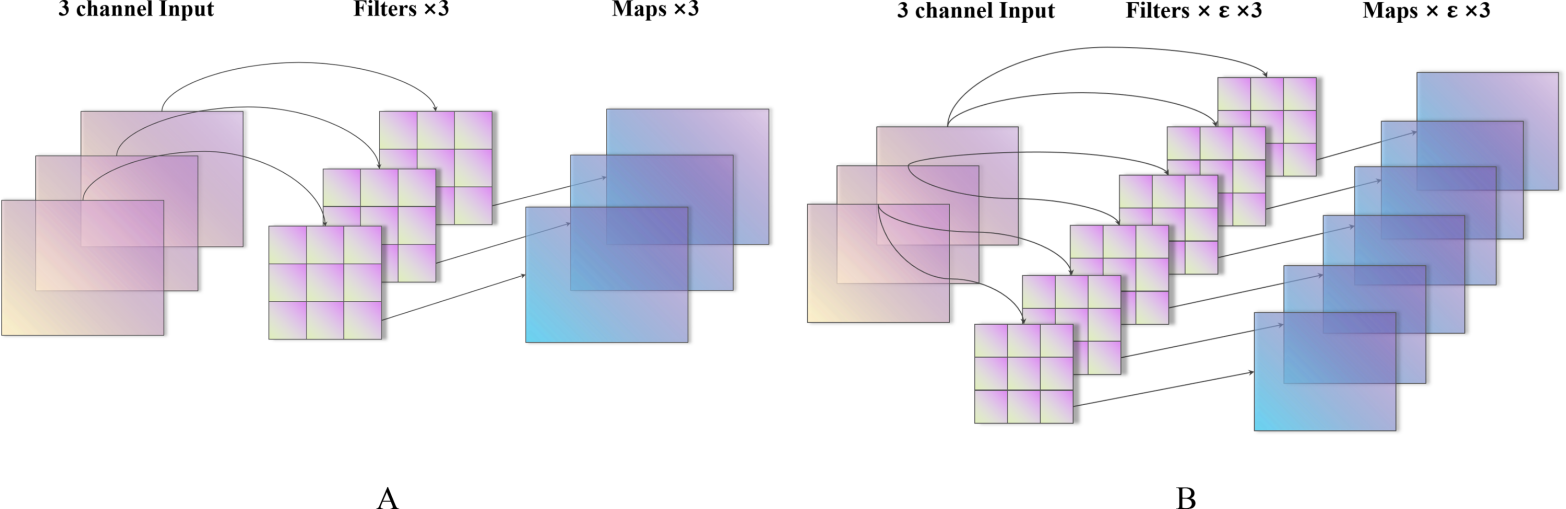
Characteristic diagram of different expansion coefficients (A–B).

### Two important factors of PlaneNet

In ‘Hyperparameter Strategy on CIFAR-10’, our experiment proves that our proposed plane network can adjust its performance flexibly according to different input size resolutions, so we discard the resolution coefficient *ρ* in MobileNetV1. The coefficient of expansion is introduced to increase the dimensionality with the depthwise convolution. Note that the size of the convolution kernel at this time is not 1. The advantage of this is that strategy 2 is perfectly addressed. This strategy can replace the 1 × 1 convolution to increase the dimension and attain a large receptive field to extract local features (Everingham et al., 2015). The details are as follows.

#### Expansion coefficient

*ɛ* corresponds to the depthwise convolution process. For example, the dimension of the input image is D_k_ × D_k_ × M (D_k_ is the size of the image, and M is the number of input channels) as shown in Fig. 3, when *ɛ* = 1, there are M D_w_ × D_w_ convolution kernels convolved with M channels and D_f_ × D_f_ × M, which is a standard depth separable convolution, output feature maps. When *ɛ* = N, there are N × M × D_w_ ×D_w_ convolution kernels, each group has N convolution kernels to convolve with each channel of the input image, and the output after convolution is D_f_  × D_f_  × N × M. N corresponds to the primary function used to change the number of channels in the production. Because of the average grouping concept, the value of N can only be positive integers; therefore, when N > 1, this is an excellent operation to increase the dimension of the input image. However, it is worth noting that although *ɛ* > 1 conducts the operation for increasing the size of the input image, there is still no step of addition or multiplication on the channel dimension, which is entirely different from the traditional convolution. The numbers of parameters and Madds of conventional convolution are, respectively,

(4) \begin{eqnarray*}{D}_{w}^{2}{\times N}^{2}\times M\end{eqnarray*}

(5) \begin{eqnarray*}{D}_{f}{\times D}_{f}\times {D}_{w}^{2}\times {N}^{\text{2}}\times M\end{eqnarray*}

The numbers of separable convolution parameters and Madds are, respectively,

(6) \begin{eqnarray*}{D}_{w}^{2}\times N\times M\end{eqnarray*}

(7) \begin{eqnarray*}{D}_{f}{\times D}_{f}\times {D}_{w}^{2}\times N\times M.\end{eqnarray*}

It is evident that when Madds reaches the same dimension, the standard convolution operation (even a 1 × 1 convolution kernel) is N times that of the separable convolution.

#### Width factor *α*

Because the performance requirements for the network are not consistent in different situations, we use the width factor in MobileNetV1. In each plane bottleneck, the number of convolution kernels is multiplied by *α*, making the network more universal; different networks can be customized for various scenes. We denote the PlaneNet with width multiplier *α* as PlaneNet *α* ×. The width factor *α* can change the size and amount of calculation of plane bottlenecks. In general, more extensive networks can achieve much more powerful performance, but this is at the expense of speed. Therefore, how to measure this hyperparameter is discussed in detail in ‘Efficiency of plane bottlenecks’.

**Table 1:** The amount of Plane bottlenecks to calculate.

layers	Input_shape	Output_shape	Parameters	Madds
DwConv k_1_×k_1_ stride=s	*h*×*w*×*c*	$ \frac{h}{s} \times \frac{w}{s} \times $ (*ɛc)*	${k}_{1}^{2}$ *c*	$ \frac{{k}_{1}^{2}hw}{{s}^{2}} $ *c*
Conv 1 ×1 stride=1	$ \frac{h}{s} \times \frac{w}{s} \times $ (*ɛc)*	$ \frac{h}{s} \times \frac{w}{s} \times $ (*αc)*	*αɛc* ^2^	$ \frac{hw}{{s}^{2}} \alpha $ *c* ^2^
DwConv k_2_×k_2_ stride=1	$ \frac{h}{s} \times \frac{w}{s} \times $(*αc)*	$ \frac{h}{s} \times \frac{w}{s} \times $(*αc)*	${k}_{2}^{2}\alpha $ *c*	$ \frac{{k}_{2}^{2}hw}{{s}^{2}} \alpha $ *c*

**Table 2:** The amount of Mobilenetv2 bottlenecks to calculate.

layers	Input_shape	Output_shape	Paramters	Madds
Conv 1 ×1 stride=1	h × w × c	h × w × (*ɛ*c)	*ɛ*c^2^	hw *ɛc*^2^
DwConv k ×k stride=s	h × w × (*ɛ*c)	$ \frac{h}{s} $$\times \frac{w}{s} \times $ (*ɛ*c)	*k*^2^*ɛ*c	$ \frac{{k}^{2}hw}{{s}^{2}} $c
Conv 1 ×1 stride=1	$ \frac{h}{s} \times \frac{w}{s} \times $ (*ɛ*c)	$ \frac{h}{s} \times \frac{w}{s} \times $ (*α*c)	*αɛ*c^2^	$ \frac{hw}{{s}^{2}} \alpha {c}^{2}$

**Table 3:** Comparison of parameters and Madds. Comparison of the number of Mobilenetv2 bottlenecks variables with the number of Plane bottlenecks variables.

	Paramters		Madds	
Model	Ours	MobileNetV2	Ours	MobileNetV2
Total	$({k}_{1}^{2}+{k}_{2}^{2}\alpha )c+\alpha {c}^{2}$	*k*^2^*ɛc* + (*α* + 1)*ɛc*^2^	$ \frac{hw}{{s}^{2}} (({k}_{1}^{2}+{k}_{2}^{2}\alpha )c+\alpha {c}^{2})$	$ \frac{hw}{{s}^{2}} $
Ratio	$ \frac{({k}_{1}^{2}+{k}_{2}^{2}\alpha )c+\alpha {c}^{2}}{{k}^{2}c+(\alpha +1){c}^{2}} \approx \frac{\alpha }{\alpha +1} $		$ \frac{ \frac{hw}{{s}^{2}} (({k}_{1}^{2}+{k}_{2}^{2}\alpha )c+\alpha {c}^{2})}{ \frac{hw}{{s}^{2}} } \approx \frac{\alpha }{\alpha +{s}^{2}} $	

### Analysis of the complexity

Here, we further analyze the number of parameters and Madds used in our plane bottlenecks. Tables 1, 2 and 3 summarized the advantages of the design of plane bottlenecks by comparing them with those of classic MobileNetV2. In this case, s represents the stride, and k represents the size of the convolution kernel (in general, it is the standard square convolution kernel, that is, k_w_ = k_h_). Take the MobileNetV2 famous deep separable convolution whose convolution kernel size is k. The size of the plane bottlenecks of the convolution kernel of the first separable convolution is k_1_, and the size of the second separable convolution kernel is k_2_. Because the filters specified in most layers are the same as the input channels, we simplify the processing by using c, where padding= ’same’ and h and w are integer multiples of s for convenience.

Generally, the value of k is relatively small (*e.g.*, *k* = 3) because multiple smaller convolution kernels can replace a larger convolution kernel, and the same receptive field can be obtained. First, this can result in fewer parameters. Second, this can allow more nonlinear transformations, which increases the ability of the CNN to learn features. The value of *ɛ* is relatively small (*e.g.*, $\in \left[ 1,6 \right] \cap \in \mathrm{Z}$); and the number of convolution kernels C is generally large (*e.g.*, 160, 256 or 320), at least 1∼2 orders of magnitude larger than that of *ɛ* and K. Therefore, the theoretical parameter ratio of the two is $\approx \frac{\alpha }{\alpha +1} $ in an ideal situation. Usually, the value of *α* fluctuates around 1 (*e.g.*, 0.5,1.0 or 1.5), so the parameter ratio between the two is 0.33 to 0.60. The setting of some of the hyperparameters in a lightweight network is insufficient to make us ignore k and *ɛ*, but this is sufficient to make plane bottlenecks take advantage of their merits. Section ‘PlaneNet on semantic segmentation’ shows the plane replacing MobileNetV2 with the rest of the hyperparameters and the same network structure setting. The parameter savings of the model are up to 34.27%.

We use Madds to assess the computational efficiency. Ideally, the theoretical parameter ratio of the two is $\approx \frac{\alpha }{\alpha +4} $, so the Madds ratio of the two is 0.11∼0.27. However, in reality, C is not much more significant than *ɛ* and *α* (much greater is defined as 10^3^). According to the Table 4, the Madds saved are generally approximately 25% compared to MobileNetV2 with the same parameter architecture.

### Building network architectures

Based on the experiments in ‘Efficiency of plane bottlenecks’ and our plane bottleneck architectures, we can easily incorporate our plane bottlenecks into existing well-designed neural architectures, thus reducing the computing costs. Using the coefficient of expansion and the width multiplier, we can tweak the architecture to achieve different performance points and adjust the architecture according to the desired precision and performance trade-off.

We present PlaneNet (*α* = 1, 224 × 224), as shown in Table 4, where the number of Madds is 111M, and the number of parameters is 0.45M. Because of the advantages of the MobileNetV3 structure, we followed it. In addition, it is essential to consider the number of layers and the number of convolved keys in MobileNetV3 with our plane bottlenecks. PlaneNet is a plane bottleneck stack that is a series of progressively increasing plane bottlenecks of various channels. These plane bottlenecks are grouped into five different stages depending on the size of the input feature map. In addition to the first plane in each location, a long stride=2, all plane bottlenecks steps taken in the convolution steps have a stride of 1. Finally, the global average pooling and convolution layer transforms the feature map into 1280D feature vectors for final classification. For large-size feature maps, the expansion coefficient is 6; and for small-size feature maps, the expansion coefficient is 3. Detailed experiments on the setting of the coefficient of expansion are conducted in ‘Hyperparameter strategy on CIFAR-10’. Compared to MobileNetV3, we do not use a hard-swish nonlinearity function due to its large latency. The squeeze and excite (SE) module is also not used. The presented architecture provides a basic design for reference, although further hyperparameter tuning or automatic architecture searching-based ghost modules will further boost the performance (He, Zhang & Sun, 2017).

**Table 4:** The overall architecture of PlaneNet. In this case, P-bneck represents Plane Bottlenecks. Out indicates the number of output channels. The Stride represents the step size of the first separable convolutional layer in Plane.Classes represent the number of Classes in the dataset.

Input	Operator	*ɛ*	out	Stride
224^2^× 3	P-bneck	6	16	2
112^2^× 16	P-bneck	6	16	1
112^2^× 16	P-bneck	5	24	2
56^2^× 24	P-bneck	5	24	1
56^2^× 24	P-bneck	5	40	2
28^2^× 40	P-bneck	5	40	1
28^2^× 40	P-bneck	5	40	1
28^2^× 40	P-bneck	3	48	2
14^2^× 48	P-bneck	3	48	1
14^2^× 48	P-bneck	3	96	1
14^2^× 96	P-bneck	3	96	1
14^2^× 96	P-bneck	3	160	2
14^2^× 160	P-bneck	3	160	1
7^2^× 160	Conv 2d 1 × 1	–	1280	1
7^2^× 1280	AvgPool 7 × 7	–	–	–
1^2^× 1280	FC	–	Classes	–

In addition, we added the LeakyReLU activation function after each convolution layer. MobileNetV2 has proven that there will be greater information loss when nonlinear activation layers, such as ReLU, are used in the shallow dimensional convolutional layer. This is because the ReLU function limits its minimum value to 0. Therefore, MobileNetV2 cancels the first ReLU function and adopts a linear bottleneck design. However, as the network deepens, the number of channels generally increases (especially with the addition of the width coefficient and expansion coefficient), and the performance of MobileNetV2 can be improved by adding a nonlinear activation function in the latter part of the network. Therefore, to trade-off the information loss and performance gains, our network uses the LeakyReLU activation function to activate the nonlinearity (Kingma & Ba, 2017; Li et al., 2019). The most significant difference between this function and the ReLU function is that instead of limiting the minimum value to 0, we multiply numbers less than 0 by leaky alpha as the output. In this way, there is no need to set the activation function of each layer for the entire network, the information loss is reduced for small dimensional data, and the ability to extract features is not weak for extensive dimensional data (Maas, Hannun & Ng, 2013). Therefore, we use a general trade-off method, and the experimental results show that this design is more robust. The model design of PlaneNet is shown in Table 4.

Note the variation rule of the expansion coefficient of PlaneNet. The expansion coefficient only changes when the stride is 2 because when the stride is 2, the image size is halved by subsampling the image twice, and the small expansion coefficient has a good effect when applied to small-sized data. The strong extraction of regional features characterizes PlaneNet, but in fact, the channel depth features cannot be ignored, so a deep convolution is applied to extract the channel features again.

## Results

**Settings:** For all the experiments in this section, the back end of the training model is TensorFlow, and most of the training settings of each model are modeled following MobileNetV2. The initial learning rate is 0.045, and the learning rate decreases at a rate of 0.98 in each epoch. The SGD optimizer is used, the momentum of the SGD optimizer is set to 0.9, and an RTX 5000 ×2 is used for training. The data enhancement strategy of our training model is to randomly rotate an image within the range of 30 deg, shift an image by 20% at the horizontal position, shift an image by 20% at the upper and lower positions, cross-cut transform 20% of an image, randomly shrink an image by 20%, and perform the horizontal flipping operation on the picture at random.

### Efficiency of plane bottlenecks

For the experiment in this section, we apply width coefficient α = 1 and expansion coefficient *ɛ* = 6 to the input dimensions unless otherwise specified.

#### Hyperparameter strategy on CIFAR-10

To evaluate our plane bottlenecks, we used the CIFAR-10 dataset and replaced all the concentric layers in the VGG16 model with our proposed plane bottlenecks substitute (the pooling layer was replaced with stride = 2 plane bottlenecks). The new models were referred to as Plane-VGG16. Our training strategy is modeled after the setup of MobileNetV2, including the optimizer’s momentum, learning rate, etc. In this paper, the effects of the hyperparameters k and s on plane bottlenecks are analyzed, and then we have compared plane bottlenecks with the most advanced light-weight network models.

CIFAR-10 is a computer vision data set for pervasive object recognition that was collected by Hinton students Alex Krizhevsky and Ilya Sutskever. It contains 60,000 32 × 32 RGB color images in 10 categories. A total of 50,000 images are used as the training set, and 10,000 images are used as the testing set.

**Table 5:** The results of the combination of plane bottlenecks and two separable convolutional layers with different convolutional kernels k_1_ and k_2_ is tested on CIFAR-10 with the remaining parameters *s* = 2, *α*=1, and *ɛ* = 6. Acc. represents accuracy.

k_1_/k_2_	Parameter (M)	Madds (M)	Acc. (%)
VGG16-BN	15.0	313.5	**92.42**
1/1	9.8	129.3	22.29
1/3	9.9	130.8	89.10
1/5	9.9	132.7	90.17
3/1	10.0	133.7	90.51
3/3	10.1	135.2	**92.60**
3/5	10.1	137.0	91.54
5/1	10.4	142.6	91.31
5/3	10.4	143.6	**92.28**
5/5	10.5	145.5	91.40

##### **Analysis of the hyperparameters**.

In this section, we analyze two hyperparameters, including k for controlling the perception fields and s for the kernel size of the linear operations of the feature mappings (that is, the size of the deep convolution filter). The impacts of these two parameters were tested on the VGG16 model architecture. First, we set {s = 2, *α*=1, *ɛ*=6} and correct and adjust the 9 sets of k_1_ and k_2_ combinations in {1, 3, 5}. The size of the plane bottlenecks of the convolution kernel of the first separable convolution is k_1,_d the size of the second separable convolution kernel is k_2_. We list the results on the CIFAR-10 verification set in Table 5. As the table shows, the experimental k_1_ = k_2_ = 3 is much better than the small or large ones in the convolution kernel. This is because the 1 × 1 kernel cannot introduce the feature information of the neighborhood plane in the feature map, and the small convolution kernel uses too much local information instead of global information. The combination of larger cores, such as {{k_1_=3, k_2_=5}, { k_1_=5, k_2_=5}}, can lead to overfitting and the failure to extract detailed local features. Therefore, to improve the effectiveness and efficiency of the experiment, we adopted the combination of k_1_=k_2_=3 in the following experiment.

After studying the convolution kernel sizes that are used in the proposed plane bottlenecks summarized, our remaining hyperparameters are set to {*α*=1, *ɛ*=6, k_1_=k_2_=3} and the hyperparameter s is set in the range of {2, 3, 4, 5, 6}. S is directly related to the computational costs of the obtained network. As analyzed in ‘The importance analysis of 1 × 1 convolution’ the larger s is, the lower the number of parameters and the computational costs. As the results in Table 6 show, when we increase s, Madds significantly decreases. The accuracy gradually decreases, which is expected because the larger the stride of the convolution kernel, the coarser the extracted local features. Notably, when *s* = 2, our algorithm is even slightly better than the original model, but the number of Madds is only 43.12% of that of VGG16, which shows the superiority of plane bottlenecks in this paper. The constant number of parameters and the rapidly decreasing number of Madds prove the correctness of our derivations in Tables 5, 6 and 7.

**Table 6:** The results of the experiment of the first separable convolution layer of plane bottlenecks on different convolution kernel step sizes s is conducted on CIFAR-10 with the remaining parameters k_1_=k_2_=3, *α*=1, and *ɛ*=6. Acc. represents accuracy.

s	Params (M)	Madds (M)	Acc. (%)
VGG16-BN	15.0	313.5	92.42
2	10.1	135.2	**92.60**
3	10.1	41.1	89.56
4	10.1	19.3	86.19
5	10.1	18.1	84.73
6	10.1	14.9	83.25

**Table 7:** The results of the experiment on the combination of the plane bottleneck width coefficient and expansion coefficient on CIFAR-10 with the remaining parameters k_1_=k_2_=3 and *s* = 2.

Model	Params (M)	Madds (M)	Acc. (%)
VGG16-BN	15.01	313.47	**92.42**
0.5/1	0.44	6.66	85.73
0.5/2	0.87	12.49	88.39
0.5/3	1.30	18.32	90.02
0.5/4	1.72	24.15	89.98
0.5/5	2.15	29.99	90.18
0.5/6	2.57	35.82	**90.82**
1.0/1	1.71	23.90	88.54
1.0/2	3.37	46.16	90.64
1.0/3	5.04	68.41	91.88
1.0/4	6.71	90.67	91.90
1.0/5	8.38	112.92	92.18
1.0/6	10.05	135.18	**92.60**
1.5/1	3.78	51.76	89.23
1.5/2	7.51	101.76	91.75
1.5/3	11.24	150.35	91.72
1.5/4	14.97	199.64	**92.82**
1.5/5	18.70	248.94	**92.79**
1.5/6	22.42	298.23	**93.1**

#### Compared with MobileNetV2 on Caltech-101

Caltech-101 consists of images with objects belonging to 101 classes, plus one background clutter class. Each image is labeled with a single object. Each class contains approximately 40 to 800 images or a total of approximately 9k images. The images have variable sizes, with typical edge lengths of 200–300 px. To study the performance of the classification task, we removed the background clutter in images and ended up with 101 classes. Since the data size is not fixed, we specifically conducted experiments with three different input sizes, namely, 192 × 192, 224 × 224, and 256 × 256. In contrast to training large models, we use fewer regularization and data augmentation techniques because small models have less trouble with overfitting. However, to be fair, we have performed experiments using data enhancement.

**Figure 4:**
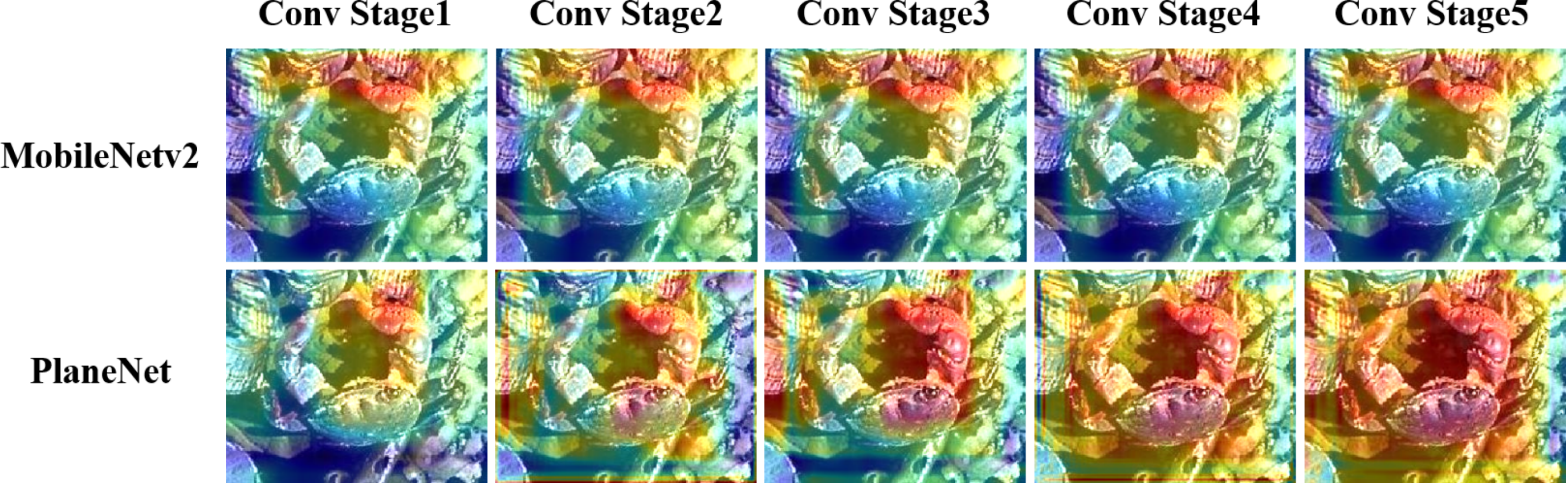
Feature extraction comparison. Conv stage i represents the Grad-CAM of the convolution layer before convolution layer i + 1 (stride=2). When *i* = 5, it is the Grad-CAM mapping of the convolution layer before the classification layer.

Next, we use plane bottlenecks to customize our MobileNetV2 algorithm. We replace plane bottlenecks with plane bottlenecks, denoted as Plane-MobileNetV2; and use the Caltech-101 data set for our experiments. The standard MobileNetV2 has approximately 3.4M parameters and 300M Madds. MobileNetV2 is based on TensorFlow open-source code. To better study the substantial differences between replacing bulk, we remove the classification layer when counting the numbers of parameters and Madds. To make a fair comparison, settings such as the optimizer and the learning rate remain the same as those set in ‘Hyperparameter Strategy on CIFAR-10’. Figure 4 shows the performances of MobileNetV2 and Plane-MobileNetV2 on the Caltech-101 dataset for different convolution stages (divided by the stride of 2). Obviously, in the first two stages, the network extracts the features of the crab’s right pincer; but from the third stage, Plane-MobileNetV2 gradually extracts the remaining features, such as the crab’s back and left pincer. However, these features are not well utilized for MobileNetV2. From this, we can see that plane bottlenecks can extract and gradually expand the features of a larger receptive field for local planar regions.

**Figure 5:**
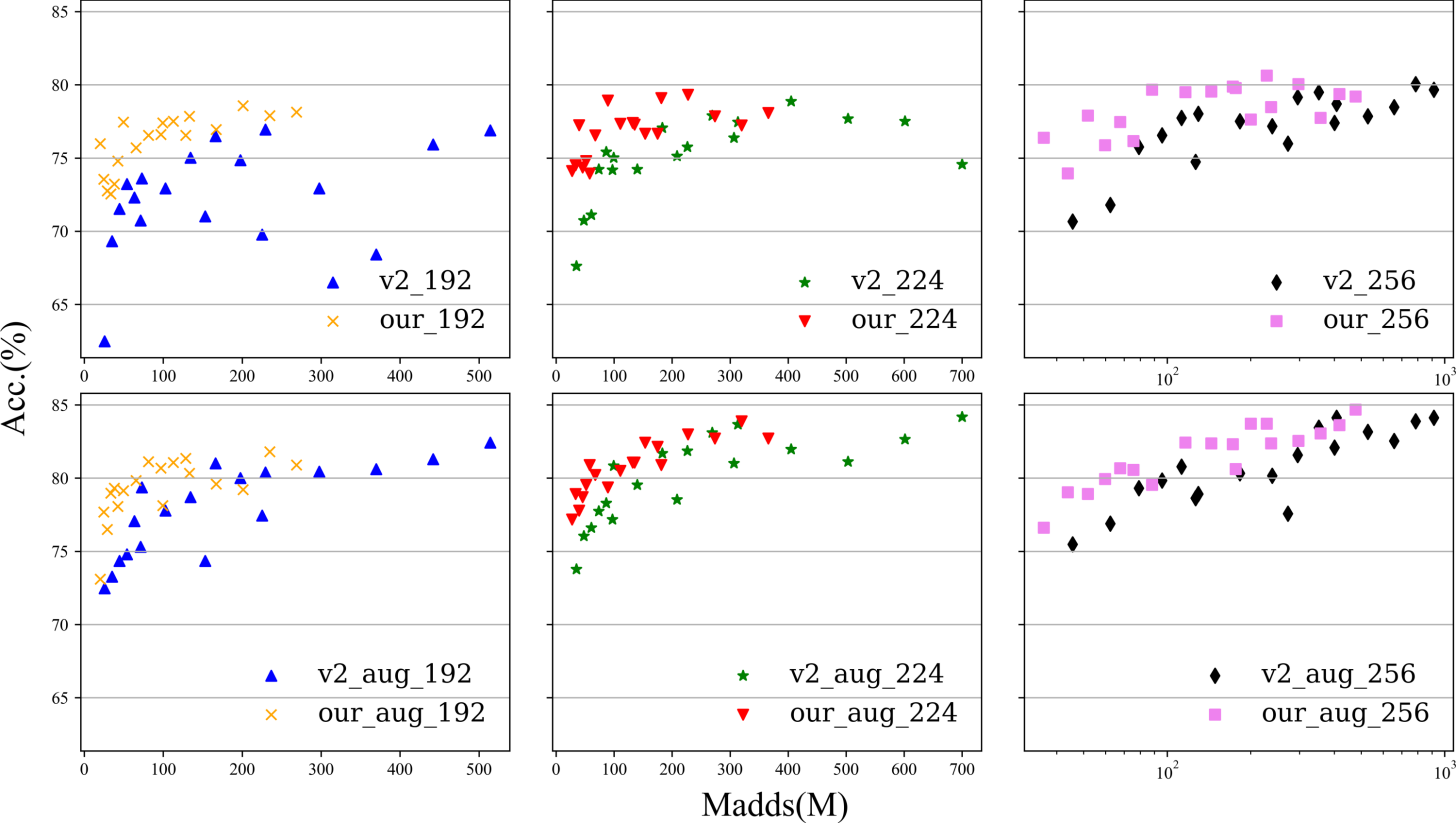
The experimental performance of the model with data enhancement and without data enhancement is tested at 192, 224, and 256 sizes. The first row is the experiment without data enhancement, and the second row is the experiment with data enhancement.

To better show the difference, we present the results in Fig. 5, where the abscissa is Madds and the ordinate is the accuracy. The results are based on experiments with images sized 192  × 192, 224  × 224, and 256  × 256. For each size, 18 groups of combinations of *α* (0.5, 1.0, and 1.5) and *ɛ* (1, 2, 3, 4, 5, and 6) were conducted. More detailed data are given in the appendix. Overall, the number of Madds of our Plane-MobileNetV2 is approximately 52.30%∼78.83% that of MobileNetV2 in the same setting, but our accuracy is higher than that of MobileNetV2 in most of the width and expansion coefficient settings. In comparison, our method achieves significantly better performance. More details are given in the appendix.

From the appendix, it is interesting to note that regardless of whether we are analyzing the data-enhanced or strengthened experiment, some common phenomena can be found: for a small-sized input image, the expansion coefficient for 3 can reach almost the same accuracy as that for 5 or 6; and for a large-sized input image, the expansion coefficient is the highest because the accuracy is almost the highest. These two interesting phenomena have constructive significance to guide us to construct PlaneNet.

### PlaneNet on image classification

To verify the performance of PlaneNet on the ImageNet datasets, we conducted experiments on the ImageNetTE and ImageNetWoof classification tasks (Bell & Koren, 2007; Berg, Deng & Fei-Fei, 2010; Breiman, 2001). ImageNetTE is a subset of 10 easily classified classes from ImageNet (trench, English springer, cassette player, chain saw, church, French horn, garbage truck, gas pump, golf ball, and parachute). ImageNetWoof is a subset of 10 classes from ImageNet that are not easy to classify since they are all dog breeds. The breeds are Australian terrier, border terrier, Samoyed, beagle, Shih Tzu, English foxhound, Rhodesian ridgeback, dingo, golden retriever, and old English sheepdog (Cireçsan, Meier & Schmidhuber, 2012; Cireçsan et al., 2011).

We follow most of the training settings used in ‘Efficiency of plane bottlenecks’ except for a batch size of 64, and we use an L2 regularization factor of 0.01 on a single GPU. For PlaneNet, for the sake of simplicity, we set k_1_=k_2_=3 in all plane bottlenecks. We choose some lightweight networks with good performance and fast speed, including the MobileNet series (Simonyan & Zisserman, 2015; He et al., 2015; Iandola et al., 2016), ShuffleNetV2 (Szegedy et al., 2014), MnasNet (Sandler et al., 2019), and GhostNet (Howard et al., 2017), as the comparison objects. The results are shown in Table 8. These models are divided into three computational complexity ranges based on the number of Madds, namely,40-60M Madds, 110–150 M Madds, and 200–300 M Madds. The results show that in these classic light-weight networks, generally, more Madds results in higher accuracy, indicating that generally the greater the number of computing resources, the better the model performance. Within the same Madds complexity range, PlaneNet test accuracy is higher than that of other models, while the number of Madds and the number of parameters are far lower than those of other models, which proves that our model can achieve high efficiency. In the three computational complexity ranges, PlaneNet always performs better than other networks at different levels of computational complexity because PlaneNet can make more effective use of the features of the plane region (Deng et al., 2009; Deng et al., 2012).

**Table 8:** Performance of various mainstream models in classification. Acc. represents the accuracy of the model on the ImageNetTE dataset. #Acc. represents the accuracy of the model on the ImageNetWoof dataset.

Model	*Acc. (%)	#Acc. (%)	Params (M)	Madds (M)
PlaneNet 0.4×	**86.11**	77.55	**0.2**	**40**
MobileNetV1 0.25×	82.83	72.97	0.5	41
MobileNetV3-small 0.75×	85.86	78.21	2.4	44
MobileNetV2 0.35×	84.94	73.41	1.7	59
MobileNetV3-small 1.0×	86.09	**78.29**	2.9	66
PlaneNet 1.0×	**89.32**	**83.35**	**0.5**	**111**
MobileNetV2 0.6×	86.52	79.00	2.2	141
GhostNet 1.0×	88.84	82.26	5.2	141
ShuffleNetV2 1.0× (*g* = 3)	87.52	80.84	2.3	146
MobileNetV1 0.5×	85.30	73.05	1.3	149
PlaneNet 1.4×	**89.86**	**84.30**	**0.9**	**195**
MobileNetv3-large 1.0×	88.56	81.58	5.4	219
MobileNetV2 1.0×	88.23	80.40	3.4	300
MnasNet 1.0×	89.45	82.16	4.2	317
MobileNetV1 0.75×	85.65	76.53	2.6	325

The classification tasks of ImageNetTE and ImageNetWoof prove that PlaneNet model has a relatively good classification performance in each segment. To further verify the generalization ability of our model, we finally used the ILSVRC2012 (Russakovsky et al., 2015) dataset to conduct the verification experiment of the classification task. ILSVRC2012, which was created by Professor Feifei Li of Stanford University, is the computer vision dataset for the ImageNet2012 competition. It has 10, 000 categories of images and 14, 197, 122 images. Due to a large amount of data in the ILSVRC2012 data set, we did not choose the commonly used graphics card resources of personal computers or the CPU resources of simulated mobile devices for this experiment. In this experiment, we used the computer resources of 4 ×NVIDIA Tesla V100s for training. We set the batch size to 512, and we chose the same settings as above for the other experimental settings. The experimental results are shown in Table 9.

**Table 9:** Performance of various mainstream models in semantic segmentation. Acc. represents the accuracy of the model on the ILSVRC2012 datasets.

Model	Acc. (%)	Params (M)	Madds (M)
PlaneNet 0.4×	67.23	**0.2**	**40**
MobileNetV1 0.25×	50.91	0.5	41
MobileNetV3-small 0.75×	64.92	2.4	44
MobileNetV2 0.35×	61.07	1.7	59
MobileNetV3-small 1.0×	**67.45**	2.9	66
PlaneNet 1.0×	**73.26**	**0.5**	**111**
MobileNetV2 0.6×	65.31	2.2	141
GhostNet 1.0×	72.97	5.2	141
ShuffleNetV2 1.0× (*g* = 3)	68.94	2.3	146
MobileNetV1 0.5×	64.21	1.3	149
PlaneNet 1.4×	**75.12**	**0.9**	**195**
MobileNetv3-large 1.0 ×	74.84	5.4	219
MobileNetV2 1.0×	72.12	3.4	300
MnasNet 1.0×	75.05	4.2	317
MobileNetV1 0.75×	66.93	2.6	325

The experimental results show that our PlaneNet model has been benchmarked on a large scale on the ILSCRC2012 dataset, and our model has excellent performance under each Madds segment with an accuracy slightly lower than that of MobileNetV3-Small only in the 40-60M stage. However, both the numbers of model parameters and Madds are much lower than those of MobileNetV3-Small. In the other stages, our model can achieve the optimal effect in terms of accuracy and the number of parameters and Madds.

To effectively verify the actual operating effectiveness of our model, we choose four graphics cards, an RTX1060, an RTX2060S, an RTX5000, and a Titan V, which represent three environments of ordinary productivity, major productivity in the next 20 years, and high-end server productivity, respectively. Then, AMD R5-3600 and Intel I7-8750H CPUs were selected to represent the future mobile terminal and low computer resource environment, respectively, as the actual operating environment to test the operating effects of our model in different environments. The experimental results are shown in Table 10. The highest index indicates the strong real-time performance of the model. The results were clear. Within the same Madds complexity range, in various operating environments, the real-time performance and fluency of our model are better than those of other networks, which can prove that our model can be effectively applied in various environments and help mobile devices and other environments effectively apply deep learning technology. In Table 10, because of the plain structure of MobileNetV1, there are not too many elementwise operations, so the speed is very fast, but the accuracy is not high. It is not advisable to accept speed and abandon precision. Therefore, we marked the highest score except that for MobileNetV1 in bold.

**Table 10:** FPS represents Frames Per Second, which is the reciprocal of the average of the 100,000 iterations it takes to predict a single image. RTX1060 represents the FPS of the model tested on the RTX1060. RTX2060s represents the FPS of the model tested on the RTX2060s. RTX5000 represents the FPS that the model was tested on the RTX5000. TitanV represents the FPS that the model was tested on the Titan V. AMD represents the R5-3600 CPU test on the AMD platform. Intel represents the i7-8750 h CPU test on the Intel platform.

Model	RTX1060	RTX2060s	RTX5000	TitanV	AMD	Intel
PlaneNet 0.4 ×	**145**	**113**	**187**	**162**	**68**	**55**
MobileNetV1 0.25 ×	217	198	253	231	75	59
MobileNetV3-small 0.75 ×	146	107	157	154	70	57
MobileNetV2 0.35 ×	128	98	158	145	48	36
MobileNetV3-small 1.0 ×	140	108	159	146	63	50
PlaneNet 1.0 ×	**147**	**110**	**179**	**170**	41	37
MobileNetV2 0.6 ×	111	95	157	147	26	20
GhostNet 1.0 ×	85	84	118	118	36	27
ShuffleNetV2 1.0 × (*g* = 3)	134	95	145	132	**53**	**41**
MobileNetV1 0.5 ×	201	177	221	211	55	40
PlaneNet 1.4 ×	**123**	**114**	**168**	**165**	**37**	**29**
MobileNetv3-large 1.0 ×	86	86	135	117	31	23
MobileNetV2 1.0 ×	101	99	153	145	21	16
MnasNet 1.0 ×	98	101	152	140	28	21
MobileNetV1 0.75 ×	178	156	199	192	35	27

However, we know that in the era of rapid development, mobile devices play an important role in daily life. To verify the superiority of PlaneNet in the information time of mobile devices, we tested FPS on raspberry pie 4b and 2b. The CPU of raspberry pie 4b is BCM2711 Cortex-A72 (ARM v8) 64-bit SoC @1.5 GHz while 2b is BCM2836 Cortex-A7 (ARM v7) 32-bit SoC @900MHz. RAM for 4b is 4G while 2b is 1G, and the results are shown in Table 11.

**Table 11:** FPS represents Frames Per Second, which is the reciprocal of the average of the 100,000 iterations it takes to predict a single image.

Model	Inference time^b^(ms)	FPS^b^	FPS^a^
PlaneNet 0.4 ×	**0.029**	**34.17**	**9.37**
MobileNetv3-small 0.75 ×	0.033	30.73	8.67
MobileNetv2 0.35 ×	0.035	28.53	6.98
MobileNetv3-small 1.0 ×	0.036	28.02	8.64
PlaneNet 1.0 ×	0.041	24.37	5.74
MobileNetv2 0.6 ×	0.047	21.24	4.68
GhostNet 1.0 ×	0.052	19.22	4.00
ShuffleNetv2 1.0 × (*g* = 3)	**0.038**	**26.29**	**6.39**
PlaneNet 1.4 ×	**0.057**	**17.42**	**4.68**
MobileNetv3-large 1.0 ×	0.067	14.82	3.16
MobileNetv2 1.0 ×	0.067	14.93	4.28

**Notes.**

a represents test FPS on raspberry pie 2b

b represents test FPS on raspberry pie 4b.

As shown in Table 11, PlaneNet’s FPS reaches 34.17, surpassing MobileNetv3-small (28.73). For models with the same level of complexity, PlaneNet can take into account both speed and accuracy, which exceeds most networks. Therefore, when we deploy the trained weight to mobile devices, the network still has advantages in speed. Therefore, it can be proved that the performance of PlaneNet is very superior.

### PlaneNet on semantic segmentation

To verify PlaneNet’s generalization ability on other computer vision tasks, we used the PASCAL VOC 2012 dataset to conduct experiments on semantic segmentation tasks (Chen et al., 2017; Chen et al., 2018; Zhao et al., 2017). PASCAL VOC’s three main object recognition contests are classification, detection, and segmentation. For the split task, VOC 2012’s travel set contains all corresponding images from 2007-2011 while the test set only contains 2008–2011. Travel has 2913 images and 6929 objects. There are 20 categories of objects (the background is category 21).

In this section, we use the ResNet50, MnasNet, GhostNet, and MobileNet series as feature extractors to test semantic segmentation tasks on the main framework based on PSPNet. The ratio of the input image spatial resolution to the backbone output resolution is set as 16, that is, the layer with the fifth convolution step of 2 is changed to 1, and the dilation rate of all separable convolution layers after this layer is set as 2. We conducted experiments on the PASCAL VOC 2012 dataset using additional annotated images. We segmented 90% of the data as the training set and 10% as the validation set. The training loss function is the Dice loss with CE and does not use auxiliary branches for auxiliary training. The Adam optimizer with 1 = 0.9 and 2 = 0.999 is adopted. The initial learning rate is 0.001, and the learning rate is multiplied by 0.1 every 500 epochs. Padding is used to crop the images to 473  × 473. The advantage is that the images will not be distorted. We applied general data enhancement strategies such as flipping. Table 12 shows the results of each model on VOC 2012. PlaneNet has nearly the same low computational costs as MobileNetV3-Small (0.48B Madds *vs.* 0.47B Madds). However, PlaneNet’s mIOU is the highest except for that of ResNet50 (74.48% *vs.* 79.92%).

**Table 12:** The results using PASCAL VOC 2012 for semantic segmentation tasks. Model mIOU with numbers of parameters and Madds.

Backbone	mIOU (%)	Params (M)	Madds (B)
ResNet50	**79.92**	46.66	50.85
MobileNetV1 1.0 ×	69.76	8.96	7.93
MnasNet 1.0 ×	72.97	3.22	3.20
MobileNetV2 1.0 ×	71.04	2.35	2.50
MobileNetv3-Large 1.0 ×	73.99	2.94	1.57
GhostNet 1.0 ×	72.87	2.65	1.17
MobileNetV3-Small 1.0 ×	69.41	0.91	**0.47**
PlaneNet 1.0 ×	74.48	**0.37**	0.48

Finally, the mIOU evaluation index is calculated on the verification set. Similarly, for the semantic segmentation task, we also calculate the Frames Per Second (FPS) for every backbone based on PSPNet. The highest index indicates the stronger real-time performance of the model. We still used the same platform in the classification network, and we chose four graphics cards: an RTX1060, an RTX2060S, an RTX5000, and a Titan V. Then, AMD R5-3600 and Intel I7-8750H CPUs were selected to test the operating effectiveness of our model in different environments. The experimental results are shown in Table 13. Regarding the actual operating effect, although our model has a slightly lower actual operating speed than MobileNetV3-Small, our model obtained the second-highest mIOU after ResNet50 while MobileNetV3-Small obtained the lowest mIOU among the comparison models. The comprehensive comparison shows that PlaneNet has the best efficiency in actual operations and can effectively utilize the model parameters.

**Table 13:** Uses Pascal VOC 2012 for the semantic segmentation tasks. FPS represents Frames Per Second, which is the reciprocal of the average of the 100,000 iterations it takes to predict a single image. RTX1060 represents the FPS for the model that was tested on the RTX1060. RTX2060s represents the FPS of the model tested on the RTX2060s. RTX5000 represents the FPS of the model that was tested on the RTX5000. TitanV represents the FPS of the model was tested on a Titan V. AMD represents the R5-3600 CPU test on the AMD platform. Intel represents the i7-8750 h CPU test on the Intel platform.

Backbone	RTX1060	RTX2060s	RTX5000	TitanV	AMD	Intel
ResNet50	11	19	28	39	6	4
MobileNetV1 1.0 ×	23	38	37	46	20	16
MnasNet 1.0 ×	21	33	35	44	20	15
MobileNetV2 1.0 ×	23	33	35	44	19	14
MobileNetV3-Large 1.0 ×	24	35	36	45	25	19
GhostNet 1.0 ×	25	31	37	44	27	21
MobileNetV3-Small 1.0 ×	29	39	**40**	46	**40**	**34**
PlaneNet 1.0 ×	**29**	**39**	38	**46**	33	27

## Conclusions

To reduce the computational costs and improve the accuracy of a deep neural network, a new plane bottleneck module is proposed to construct an efficient neural network structure. The basic plane module is divided into three parts. The first part increases the dimensions using separable convolution, which effectively reduces the number of 1 × 1 convolutions. Then, the local features are extracted through point-by-point convolution. Finally, the plane features are obtained through separable convolution. Experiments on classification tasks and semantic segmentation tasks show that this module is a plug-and-play module that can reduce the calculation costs of the original model and obtain better performance to a certain extent. Additionally, the PlaneNet constructed using the proposed new module is superior to the most advanced portable neural networks in both efficiency and accuracy. The original data are given in Tables 14, 15 and 16.

**Table 14:** Comparing the numbers of parameters and Madds of MobileNetV2 and PlaneNet. The corresponding size of * is 224 × 224, the corresponding size of ** is 192 × 192 and the corresponding size of *** is 256 × 256.

*α*/*ɛ*	Parameters (M)*		Madds (M)*		Madds (M)**		Madds (M)***	
	Mobilev2	Ours	Mobilev2	Ours	Mobilev2	Ours	Mobilev2	Ours
0.5/1	0.30	0.27	34.91	27.52	25.65	20.22	45.59	35.95
0.5/2	0.38	0.32	47.74	33.60	35.07	24.69	62.35	43.89
0.5/3	0.46	0.37	60.57	39.68	44.50	29.15	79.11	51.83
0.5/4	0.54	0.42	73.40	45.76	53.93	33.62	95.87	59.77
0.5/5	0.62	0.46	86.23	51.84	63.35	38.08	112.63	67.71
0.5/6	0.71	0.51	99.06	57.92	72.78	42.55	129.39	75.65
1.0/1	0.73	0.62	96.88	67.56	71.18	49.64	126.54	88.24
1.0/2	1.03	0.80	139.97	89.03	102.84	65.41	182.82	116.28
1.0/3	1.34	0.97	183.06	110.49	134.50	81.18	239.10	144.32
1.0/4	1.65	1.15	226.16	131.96	166.16	96.95	295.39	172.35
1.0/5	1.95	1.32	269.25	153.42	197.81	112.72	351.67	200.39
1.0/6	2.26	1.50	313.34	174.89	229.47	128.49	407.95	228.43
1.5/1	1.62	1.36	208.46	135.17	153.15	99.31	272.27	176.55
1.5/2	2.29	1.74	306.73	181.33	225.35	133.22	400.63	236.84
1.5/3	2.97	2.13	405.00	227.49	297.55	167.14	528.98	297.13
1.5/4	3.64	2.51	503.27	273.64	369.75	201.05	657.33	357.42
1.5/5	4.31	2.90	601.54	319.81	441.95	234.96	785.69	417.72
1.5/6	4.99	3.28	699.81	365.97	514.15	268.88	914.04	478.01

**Table 15:** Under different sizes, the precision comparison of MobileNetV2 and PlaneNet after data enhancement. Red denotes that a measure is better than the other measure. “Our acc.” refers to PlaneNet, and M.V2 refers to MobileNetV2.

*α*/*ɛ*	Input_size=(192 × 192)		Input_size=(224 × 224)		Input_size=(256 × 256)	
	Our acc. (%)	M.v2 acc. (%)	Our acc. (%)	M.v2 acc. (%)	Our acc. (%)	M.v2 acc. (%)
0.5/1	73.10	72.48	77.17	73.78	76.61	75.48
0.5/2	77.68	73.27	78.92	76.04	79.03	76.89
0.5/3	76.49	74.35	77.79	76.61	78.92	79.32
0.5/4	78.98	74.80	78.70	77.74	79.94	79.83
0.5/5	79.32	77.06	79.54	78.30	80.67	80.79
0.5/6	78.07	79.37	80.90	80.84	80.56	78.92
1.0/1	79.15	75.31	80.22	77.17	79.54	78.64
1.0/2	79.83	77.79	79.37	79.54	82.42	80.33
1.0/3	81.12	78.70	80.50	81.69	82.37	80.16
1.0/4	80.68	81.01	81.07	81.86	82.31	81.58
1.0/5	81.07	80.00	82.42	83.10	83.72	83.44
1.0/6	81.35	80.39	82.15	83.67	83.72	84.12
1.5/1	78.13	74.35	81.07	78.53	80.62	77.57
1.5/2	80.33	77.45	80.90	81.01	82.37	82.09
1.5/3	79.60	80.45	82.99	81.97	82.54	83.16
1.5/4	79.21	80.62	82.71	81.12	83.05	82.54
1.5/5	81.80	81.29	83.89	82.65	83.61	83.89
1.5/6	80.90	82.42	82.71	84.18	84.68	84.12

**Table 16:** For different sizes, the accuracy comparison between MobileNetV2 and PlaneNet without data enhancement. Red denotes that a measure is better than the other measure. “Our acc.” refers to PlaneNet, and M.V2 refers to MobileNetV2.

*α*/*ɛ*	Input_size=(192 × 192)		Input_size=(224 × 224)		Input_size=(256 × 256)	
	Our acc. (%)	M.v2 acc. (%)	Our acc. (%)	M.v2 acc. (%)	Our acc. (%)	M.v2 acc. (%)
0.5/1	75.98	62.48	74.12	67.62	76.38	70.67
0.5/2	73.55	69.32	74.51	70.73	73.95	71.80
0.5/3	72.76	71.52	77.25	71.12	77.90	75.76
0.5/4	72.54	73.22	74.35	74.23	75.87	76.55
0.5/5	73.22	72.31	74.80	75.42	77.45	77.74
0.5/6	74.80	73.61	73.95	75.02	76.15	78.02
1.0/1	77.45	70.73	76.55	74.18	79.66	74.74
1.0/2	75.70	72.93	78.92	74.23	79.49	77.51
1.0/3	76.55	75.02	77.34	77.06	79.54	77.17
1.0/4	76.61	76.49	77.40	75.76	79.88	79.15
1.0/5	77.51	74.85	76.66	77.90	77.62	79.49
1.0/6	76.55	76.94	76.66	77.45	80.62	78.70
1.5/1	77.40	71.01	77.28	75.14	79.77	75.98
1.5/2	77.85	69.77	79.09	76.38	78.47	77.40
1.5/3	76.94	72.93	79.32	78.87	80.05	77.85
1.5/4	78.58	68.41	77.85	77.68	77.74	78.47
1.5/5	77.90	75.93	77.23	77.51	79.37	80.05
1.5/6	78.13	76.89	78.08	74.57	79.20	79.66

## Additional Information and Declarations

### Data Availability

The following information was supplied regarding data availability: The data was tested by using the datasets available here: - Caltech101:
http://www.vision.caltech.edu/Image_Datasets/Caltech101/. - ImageNetTE, ImageNetWoof:
https://image-net.org/. - VOC2012: https://pjreddie.com/projects/pascal-voc-dataset-mirror. - Cifar10:
http://www.cs.toronto.edu/ kriz/cifar.html. - The PlaneNet code is available at GitHub: https://github.com/LinB203/planenet.
